# Electrophysiological differences between Hirayama disease, amyotrophic lateral sclerosis and cervical spondylotic amyotrophy

**DOI:** 10.1186/1471-2474-15-349

**Published:** 2014-10-16

**Authors:** Xiang Jin, Jian-Yuan Jiang, Fei-Zhou Lu, Xin-Lei Xia, Li-Xun Wang, Chao-Jun Zheng

**Affiliations:** Department of Orthopedics, Huashan Hospital, Fudan University, Shanghai, 200040 China

**Keywords:** Hirayama disease, Electrophysiological differentiation, Amyotrophic lateral aclerosis, Spondylotic amyotrophy, Action potential

## Abstract

**Background:**

Hirayama disease (HD), amyotrophic lateral sclerosis (ALS) or cervical spondylotic amyotrophy (CSA) may result in atrophy of intrinsic hand and forearm muscles. The incidence of HD is low, and it is rarely encountered in the clinical setting. Consequently, HD is often misdiagnosed as ALS or CSA. It is important to differentiate these diseases because HD is caused by a benign focal lesion that is limited to the upper limbs.

**Methods:**

The thenar and hypothenar compound muscle action potential (CMAP) amplitude of the upper limbs of 100 HD, 97 ALS and 32 CSA cases were reviewed; 35 healthy individuals were included as controls. Seventy-eight percent, 38% and 69% of patients with HD, ALS or CSA had unilateral involvement; the remaining patients were affected bilaterally. Thenar and hypothenar CMAP amplitude evoked by ulnar stimulation was compared with CMAP evoked by median stimulation.

**Results:**

The ulnar/median CMAP ratio was found to be lower in HD (0.55 ± 0.41, P < 0.0001), higher in ALS (2.28 ± 1.15, P < 0.0001) and no different in CSA (1.21 ± 0.53, P > 0.05) compared with the normal range from previous studies (0.89-1.60) and with the healthy controls (1.15 ± 0.23). Conduction velocities of the sensory and motor nerves, the amplitude of the sensory nerve action potential, and the CMAP amplitude of the unaffected limb were all normal.

**Conclusions:**

The hand muscles were differentially affected between patients with HD, ALS and CSA. The ulnar/median CMAP ratio could be used to distinguish these three diseases.

## Background

Hirayama disease (HD), also known as juvenile muscular atrophy of the distal upper extremity, was first reported by Hirayama et al. in 1959 as a benign focal motor neuron disease characterized by unilateral atrophy of the forearm ulnar muscle[1]. HD primarily affects males (male:female ratio of 20:1) in their early years (predominantly in the range of 15-25 years of age). Less than 1500 patients with HD were reported in the literature, and these cases were mostly distributed in Japan, other Asian countries, Europe and North America.

The contribution of family history was rarely observed in HD. In addition, in most patients, the disease stabilizes 1-5 years after an insidious onset. However, in rare cases, further amyotrophy progression has been reported. The pathogenesis of HD remains obscure. Spinal cord ischemia caused by cervical flexion, autoimmune diseases, idiopathic disorders and gene mutations were proposed as causes of the disease. The clinical features of HD are similar to those of amyotrophic lateral sclerosis (ALS) and cervical spondylotic amyotrophy (CSA). ALS manifests by the progressive and malignant degeneration of motor nerves. The clinical features are limb spasticity, focal or multifocal limb weakness, wasting, dysarthria, dysphagia and dysmasesis[2]. In about 40% of patients, ALS first affects the upper limbs, and limb weakness is usually asymmetric in the early stages of the disease.

In CSA, lesions to the anterior horn cells are a result of an indirect insufficient blood supply due to cervical canal stenosis, or by compression or stretching of the intra- and extramedullary vessels with movements of the cervical spine[3]. The clinical features of CSA include atrophy of the deltoid, scapular and interosseous muscles, leading to upper limb muscular atrophy. A recent review has shown that minimal or absent radicular pain or paraesthesia of the upper limbs was accompanied by spindle spondylopatic radiculapathy or myelopathy[3, 4]. CSA has been classified as proximal or distal according to the predominant site of the lesion[5]. The affected muscles are the deltoid and biceps (innervated by the C5 and C6 roots) in proximal cases, while extensor and flexor muscles of the fingers and intrinsic hand muscles (innervated by the C7-T1 roots) are found in distal cases.

The incidence of HD is low, and it is rarely encountered in the clinical setting. Consequently, HD is often misdiagnosed as ALS or CSA. It is important to differentiate these diseases because HD is caused by a benign focal lesion that is limited to the upper limbs. In contrast, ALS involves systemic muscles and may result in respiratory failure, while CSA may lead to upper motor neuron damage that could adversely affect the control of the lower limbs.

In the present study, we compared the thenar and hypothenar amplitudes of the upper limbs in patients with HD, ALS or CSA, as well as in healthy controls[6]. Such detailed examination could enable the creation of an index for the differentiation of these similar diseases.

## Methods

Results from 229 patients who underwent thenar and hypothenar compound muscle action potential (CMAP) amplitude examinations between January 2007 and January 2013 at the Huashan Hospital, Fudan University (Shanghai, China), were retrospectively reviewed. The project was approved by the Human Ethics Committee of the Huashan Hospital, Fudan University, China, and the need for individual consent was waived since this was a retrospective study. One hundred of these cases were diagnosed as HD, 97 as ALS, and 32 as CSA. For comparison, 35 healthy people who underwent CMAP examination as part of their routine complete check-up were included. Subjects with any relevant diseases or systemic conditions were excluded. All participants underwent electromyography of the thenar and hypothenar muscles as conventional diagnosis method.

Criteria for diagnosis of HD were: 1) onset before 25 years of age; 2) unilateral or bilateral weakness of the distal upper limbs, accompanied by distal amyotrophy of the hand and forearm; 3) unaffected lower limbs; 4) unilateral or asymmetric; and 5) stabilization of the disease after an initial slow progression over several years[7, 8]. In addition, lower cervical compression resulting from anterior shifting of the posterior dura, and crescent abnormality posterior to the dura were evaluated by MRI under neck flexion[9, 10].

ALS was diagnosed according to the El Escorial criteria: 1) progressive muscle weakness and wasting; 2) fasciculation and pyramidal sign; and 3) absence of sensory deficit or sphincter dysfunction[11].

The criteria for CSA diagnosis were: 1) unilateral or bilateral weakness of the distal upper limbs accompanied by distal amyotrophy of the hand and forearm; 2) minimal or absence of sensory deficit of the upper limbs; 3) hyperreflexia or normal function of the lower limbs; 4) minimal or absence of radiative pain of the upper limbs; and 5) a history of cervical spondylosis[12].

Exclusion criteria were: 1) history of syringomyelia, spinal cord tumor or abnormalities of the cervical vertebrae; 2) focal or multifocal neuropathy; 3) brachial plexus lesion; 4) congenital muscular dystrophy; or 5) injury or infection at presentation.

During motor nerve examination, thenar and hypothenar CMAP values were recorded at the abductor pollicis brevis (APB) and abductor digiti minimi (ADM) in response to stimulation of the median and ulnar nerves, respectively. The median nerve was stimulated at the wrist and elbow. The ulnar nerve was stimulated at the wrist, above and below the elbow (10 cm distance between above and below the elbow, under elbow flexion at 90°). A surface electrode was placed at the belly of the muscles (thenar and hypothenar) as the active or test electrode, and at the tendon as the reference electrode. In sensory nerve examination, the median and ulnar nerves were stimulated at the wrist. By contrast, the sensory nerve action potentials (SNAP) were recorded at the index and little fingers with the active electrode placed at the metacarpophalangeal joints, and the reference electrode placed 4 cm distal to the active electrode.

We measured CMAP and SNAP from the affected and unaffected limbs of the patients with unilateral limb involvement, and the conduction velocities of the motor and sensory nerves. For those participants who had bilateral upper limbs involvement, the amplitudes of the most affected hand were recorded. In the healthy group, nerve conduction studies were all performed in the left hand. For each parameter, we calculated the ulnar/median (U/M) ratios. A U/M CMAP amplitude ratio of <0.6 or >1.7 was regarded as abnormal[13, 14].

Student’s t-tests and Pearson’s rank correlation coefficients were used for statistical analysis. A P-value <0.05 was considered statistically significant.

## Results

Demographic data are presented in Table 1. Seven of the 97 patients with ALS (3 with carpal tunnel syndrome, and 4 with multiple peripheral neuropathy), and 2 of the 32 patients with CSA (1 with cubital tunnel syndrome, and 1 with carpal tunnel syndrome) were excluded from the study due to complications. Among the included patients, 78 of the 100 (78.0%) patients with HD, 37 of the 90 (41.1%) patients with ALS, and 22 of the 32 (68.8%) patients with CSA were affected unilaterally, while the remaining cases were affected bilaterally.

**Table 1:** **Demographic characteristics of patients with HD**, **ALS or CSA**

	HD	ALS	CSA	Normal controls
N	100	90	30	35
Male/Female	95/5	52/38	19/11	25/10
Mean onset age (years)	19.8 (14-27)	56.4 (36-72)	54.8 (42-76)	27.2(20-41)
Mean duration (months)	14.5 (6-30)	24.8 (3-48)	26.2 (3-60)	
Unilateral upper limb affected	78	37	22	
Bilateral upper limbs affected	22	53	8	

HD, Hirayama disease; ALS, amyotrophic lateral sclerosis; CSA, cervical spondylotic amyotrophy.

Nerve conduction was successfully recorded for the sensory and motor nerves in all patients (Table 2). We observed significantly lower ulnar CMAP amplitudes and slightly lower median CMAP amplitudes for the affected limbs in patients with HD, resulting in a mean U/M CMAP ratio of 0.55 ± 0.41, which was significantly lower than the previously reported normal ratio of 0.89-1.60[13, 14], or the ratio of 1.15 ± 0.23 observed in the healthy controls. The difference between the median and the median ulnar CMAP amplitudes was 4.5 ± 0.3 mV (range: -3.0 - 12.5 mV). An abnormal median CMAP amplitude (<6.0 mV) was found in 23 patients (23%) and an abnormal ulnar CMAP amplitude (<5.5 mV) was found in 83 patients (83%) of the HD group.

**Table 2:** **Motor nerve conduction tests in patients with HD**, **ALS or CSA**

		Median nerve		Ulnar nerve		U/M ratio	
		Amplitude (mV)	CV (m/s)	Amplitude (mV)	CV (m/s)	Amplitude	CV
HD	Affected limb	8.2 ± 3.9^&^	59.3 ± 4.3	3.2 ± 2.6^&^	60.3 ± 5.5	0.55 ± 0.41^&^	1.02 ± 0.11
	Healthy limb	10.3 ± 2.4	60.2 ± 3.5	9.0 ± 1.9	59.3 ± 4.2	0.98 ± 0.22	1.03 ± 0.11
ALS	Affected limb	2.3 ± 2.2*^&^	55.5 ± 4.5*^&^	4.8 ± 3.5*^&^	56.6 ± 6.0*^&^	2.28 ± 1.25*^&^	1.10 ± 0.18
	Healthy limb	7.9 ± 2.4^#^	54.8 ± 3.9*	8.4 ± 2.4	55.2 ± 4.2^#^	1.12 ± 0.32	1.04 ± 0.14
CSA	Affected limb	4.5 ± 3.1*^&^	57.4 ± 5.2	4.9 ± 3.1*^&^	55.1 ± 4.1*^&^	1.21 ± 0.53*	0.95 ± 0.13
	Healthy limb	8.2 ± 2.0^#^	57.2 ± 3.2*	7.8 ± 1.8^#^	55.2 ± 4.3*	0.97 ± 0.34	0.93 ± 0.17
Normal controls		9.0 ± 1.5	59.8 ± 3.1	9.5 ± 1.8	60.2 ± 3.2	1.15 ± 0.23	1.01 ± 0.12

*P < 0.0001, the affected (healthy) limbs of patients with ALS or CSA vs. the affected (healthy) limbs of patients with HD.

^#^P < 0.001, the affected (healthy) limbs of patients with ALS or CSA vs. the affected (healthy) limbs of patients with HD.

^&^P < 0.0001, the affected limbs of patients with HD, ALS or CSA patients vs. the limbs of normal controls.

HD, Hirayama disease; ALS, amyotrophic lateral sclerosis; CSA, cervical spondylotic amyotrophy; CV, conduction velocities; U/M, ulnar/median.

We also observed slightly lower ulnar and significantly lower median CMAP amplitudes in the affected limbs of patients with ALS, resulting in a mean U/M CMAP ratio of 2.28 ± 1.25, which was significantly higher than the normal ratio. The difference between the median and the median ulnar CMAP amplitudes was -1.3 ± 1.6 mV (range -6.7 - 4.5 mV). In the ALS group, we found abnormal median CMAP amplitude in 66 (73%) patients, and abnormal ulnar CMAP amplitude in 48 (54%) patients.

Slightly decreased ulnar and median CMAP amplitudes were found in the affected limbs of patients with CSA, resulting in a mean U/M CMAP ratio of 1.21 ± 0.53, which was comparable to the normal ratio. The difference between the median and the median ulnar CMAP amplitudes was 0.4 ± 1.6 mV (range -2.7 - 4.6 mV). Abnormal median CMAP amplitude was found in 16 patients (53%), and abnormal ulnar CMAP amplitude was found in 17 patients (57%) in the CSA group. Additionally, significant differences were found between the U/M CMAP ratios of the three diseases (P < 0.0001), as well as between patients with HD and controls (P < 0.0001), and between patients with ALS and controls (P < 0.0001).

The motor nerve conduction velocities (CV) was marginally higher than the lower limit of the normal range in patients with ALS, but was found to be normal in patients with HD or CSA. Additionally, lower ulnar median CVs in 6 (0.07) patients with ALS and lower median CVs in 8 (0.10) patients with ALS were observed. The extent of these lower values was very similar, and the U/M CV ratio was about 1.0. A similar ratio was found in patients with HD or CSA.

The U/M CMAP ratios are presented in Table 3. Patients with ALS showed a positive correlation between the ulnar CMAP amplitude and the ulnar motor nerve CV (R = 0.452, P = 0.002). Such a correlation was absent in patients with HD or CSA. We found sixty-six affected limbs with an abnormally low ratio (<0.6) (62 patients with HD, 3 patients with ALS and 1 patient with CSA). Forty-five affected limbs exhibited an abnormally high ratio (>1.7) (10 patients with HD, 33 patients with ALS and 2 patients with CSA). Moreover, normal CMAP and motor nerve CVs were observed for all unaffected limbs.

**Table 3:** **U/M CMAP ratio in patients with HD, ALS or CSA**

		U/M CMAP ratio		
		<0.6	0.6-1.7	>1.7
HD	Affected limb	62	28	10
	Healthy limb	0	78	0
ALS	Affected limb	3	54	33
	Healthy limb	0	37	0
CSA	Affected limb	1	27	2
	Healthy limb	0	22	0
Normal controls	Healthy limb	0	35	0

HD, Hirayama disease; ALS, amyotrophic lateral sclerosis; CSA, cervical spondylotic amyotrophy; CV, conduction velocities; U/M, ulnar/median; CMAP, compound muscle action potential.

The results of the sensory nerve conduction tests are presented in Table 4. We found normal SNAP amplitudes and sensory nerve CVs in all patients. Median and ulnar SNAP were different between controls and all three disease groups (P < 0.0001), but the U/M ratio was similar between the four groups.

**Table 4:** **Sensory nerve conduction tests in patients with HD**, **ALS or CSA**

		Median nerve		Ulnar nerve		U/M ratio	
		Amplitude (μV)	CV (m/s)	Amplitude (μV)	CV (m/s)	Amplitude	CV
HD	Affected limb	50.1 ± 13.2^&^	62.1 ± 6.4^&^	42.5 ± 13.7^&^	60.1 ± 5.5	0.9 ± 0.4	1.0 ± 0.1
	Healthy limb	50.4 ± 12.1	61.2 ± 6.4	44.9 ± 11.4	60.5 ± 6.2	0.9 ± 0.3	1.0 ± 0.1
ALS	Affected limb	31.7 ± 14.3*^&^	57.5 ± 8.3*^&^	31.9 ± 13.4*^&^	56.6 ± 6.9*^&^	0.9 ± 0.3	0.9 ± 0.1
	Healthy limb	37.6 ± 12.3*	57.8 ± 5.9*	34.8 ± 13.1*	56.2 ± 5.2*	0.9 ± 0.3	1.0 ± 0.1
CSA	Affected limb	38.9 ± 13.2*^&^	57.6 ± 5.7*^&^	36.5 ± 11.3*^&^	57.1 ± 4.5*^&^	0.9 ± 0.2	0.9 ± 0.1
	Healthy limb	38.2 ± 11.8*	57.2 ± 4.3*	37.5 ± 11.7*	57.2 ± 4.3*	0.9 ± 0.3	0.9 ± 0.2
Normal controls		45.6 ± 15.2	60.7 ± 5.2	38.1 ± 11.1	60.5 ± 5.2	0.9 ± 0.2	0.9 ± 0.1

*P < 0.0001, the affected (healthy) limbs of patients with ALS or CSA patients vs. the affected (healthy) limbs of patients with HD.

^&^P < 0.0001, the affected limbs of patients with HD, ALS or CSA vs. the limbs of normal control.

HD, Hirayama disease; ALS, amyotrophic lateral sclerosis; CSA, cervical spondylotic amyotrophy; CV, conduction velocities; U/M, ulnar/median.

## Discussion

Compared with healthy subjects, we observed that the U/M CMAP ratio was lower in patients with HD, higher in patients with ALS, and in the normal range for patients with CSA. Although both thenar and hypothenar muscles are innervated by the C8/T1 segments, different patterns of muscle weakness and wasting were observed between HD, ALS and CSA. Compared with thenar muscles, more severe influences on hypothenar muscles were also found in patients with HD. The ADM amplitude was previously reported to be marginally lower than the APB amplitude in normal subjects, which produced a U/M CMAP ratio of 0.82 (range: 0.6 - 1.7)[13, 14]. In the present study, the U/M CMAP ratio was less than 0.6 in 62 patients with HD (61%) and supported the more severe effects exhibited by the hypothenar muscle.

The thenar muscle is more vulnerable in patients with ALS, which involves greater decreases in the motor unit for APB than that seen for ADM. A mean APB-ADM amplitude difference of -1.7 mV, a mean APB/ADM CMAP ratio of 0.7 and significantly lower APB/ADM CMAP ratios have been reported in 41% of patients of a prospective study[13]. In our study, the mean difference was -1.3 mV, while the mean U/M CMAP ratio was 2.28, with a significantly higher U/M CMAP ratio found in 37% of patients. These observations confirmed the importance of the more severe involvement of the thenar muscle in patients with ALS.

The thenar and hypothenar muscles are affected to a similar extent in patients with CSA. In our study, the U/M CMAP ratio was in the normal range in 27 patients (90%). However, a mean APB-ADM amplitude difference of 2.6 mV, which was consistent with a more severe atrophy of the hypothenar muscle, was previously reported by Kuwabara et al.[13]. This discrepancy may result from sampling differences between the two studies. For example, patients with distal CSA were included in our study while Kuwabara et al.[13] excluded them.

In contrast, the hypothenar muscles are affected more severely in patients with HD. In our study, more severe effects on the hypothenar muscle were found in the affected limbs of only 3 patients with ALS (2%) and only 1 patient with CSA (3%). In addition, 94% of the affected limbs showing a U/M CMAP ratio <0.6 were found in HD. Thus, a U/M CMAP ratio <0.6 is strongly indicative of a diagnosis of HD but not of ALS. Simultaneously, more severe effects on the thenar muscle were found in the affected limbs of 10 patients with HD (22%) and 2 patients with CSA (4%). Consequently, only 74% of all the affected limbs showing a U/M CMAP ratio >1.7 was found in ALS and, importantly, this observation disqualified the U/M CMAP ratio criteria in the diagnosis of ALS.

Currently, the pathogenesis of both HD and CSA remains unclear. Two main hypotheses for understanding HD pathogenesis include the "dynamics" hypothesis of Hirayama and the "imbalanced growth" hypothesis of Toma. The former involves a circulation deficit during neck flexion, which results in degeneration of the anterior horn and neurogenic atrophy of the innervated muscles[15, 16]. The circulation deficit of the spinal anterior artery has been suggested by angiography to be a prominent factor in the degeneration process[17, 18]. The imbalanced growth of both the spinal cord and the spinal dura was proposed by Toma et al. to be a possible cause, based on the growth curves of 7 patients. It is proposed that the inflexible dura will compress the spinal cord during neck flexion, and repeated neck flexion will lead to microinjuries and finally to spinal cord atrophy[19]. Similarly, specific compression of the ventral roots in cervical spondylosis was suggested by Keegan et al. to result in separative motor neuron injury of the upper limbs in CSA[20]. An alternative explanation involves a deficiency in blood supply, and subsequent lesions of the anterior horn cells following compression[21].

The differences between HD and CSA have been less discussed. Both diseases share similar pathogenesis and similar clinical features, but they have distinct ages of onset. HD predominantly affects juveniles, while CSA mostly affects the aged population. In our study, the mean age of onset in patients with HD was 19.8 years, while it was 54.8 years in CSA. However, there remains some controversy in the literature: is HD a juvenile form of CSA or is CSA a aged form of HD[22, 23]?

## Conclusions

In conclusion, the different patterns of hand muscle atrophy indicate that HD, CSA and ALS are different diseases with their own distinct pathogenesis. Therefore, the U/M CMAP ratio could be considered as a criterion in the differential diagnosis of HD, ALS and CSA. A low U/M ratio is suggestive of HD, while a high U/M ratio is suggestive of ALS.

## Abbreviations

ADM: Abductor digiti minimi
ALS: Amyotrophic lateral sclerosis
APB: Pollicis brevis
CMAP: Compound muscle action potential
CV: Conduction velocities
CSA: Cervical spondylotic amyotrophy
HD: Hirayama disease
SNAP: Sensory nerve action potential
U/M: Ulnar/median.
